# Optimized CNT-PDMS Flexible Composite for Attachable Health-Care Device

**DOI:** 10.3390/s20164523

**Published:** 2020-08-13

**Authors:** Jian Du, Li Wang, Yanbin Shi, Feng Zhang, Shiheng Hu, Pengbo Liu, Anqing Li, Jun Chen

**Affiliations:** Advanced Micro and Nanoinstruments Center (AMNC), School of Mechanical & Automotive Engineering, Qilu University of Technology (Shandong Academy of Sciences), Jinan 250353, China; 1043117031@stu.qlu.edu.cn (J.D.); syb@qlu.edu.cn (Y.S.); 201701040032@stu.qlu.edu.cn (F.Z.); 1043118164@stu.qlu.edu.cn (S.H.); pengbo@qlu.edu.cn (P.L.); akin@qlu.edu.cn (A.L.); chenjun@qlu.edu.cn (J.C.)

**Keywords:** carbon nanotubes, CNT-PDMS composites, piezoresistance sensor, flexible strain sensors

## Abstract

The CNT-PDMS composite has been widely adopted in flexible devices due to its high elasticity, piezoresistivity, and biocompatibility. In a wide range of applications, CNT-PDMS composite sensors were used for resistive strain measurement. Accordingly, the percolation threshold 2%~4% of the CNT weight ratio in the CNT-PDMS composite was commonly selected, which is expected to achieve the optimized piezoresistive sensitivity. However, the linear range around the percolation threshold weight ratio (2%~4%) limits its application in a stable output of large strain (>20%). Therefore, comprehensive understanding of the electromechanical, mechanical, and electrical properties for the CNT-PDMS composite with different CNT weight ratios was expected. In this paper, a systematic study was conducted on the piezoresistivity, Young’s modulus, conductivity, impedance, and the cross-section morphology of different CNT weight ratios (1 to 10 wt%) of the CNT-PDMS composite material. It was experimentally observed that the piezo-resistive sensitivity of CNT-PDMS negatively correlated with the increase in the CNT weight ratio. However, the electrical conductivity, Young’s modulus, tensile strength, and the linear range of piezoresistive response of the CNT-PDMS composite positively correlated with the increase in CNT weight ratio. Furthermore, the mechanism of these phenomena was analyzed through the cross-section morphology of the CNT-PDMS composite material by using SEM imaging. From this analysis, a guideline was proposed for large strain (40%) measurement applications (e.g., motion monitoring of the human body of the finger, arm, foot, etc.), the CNT weight ratio 8 wt% was suggested to achieve the best piezoresistive sensitivity in the linear range.

## 1. Introduction

With the increasing attention to physical health, portable and flexible devices (e.g., stretchable electronic skin [1] and flexible strain sensors [2]) that can monitor psychological indicators and motions in real-time are attracting more and more interest. Among them, CNT-PDMS-based flexible sensors were commonly used to measure strain and stress caused by bending behaviors in the body. Notably, both the strain and the stress of the human body changed with large scale magnitudes. For example, the wrist pulse caused strain change within 2% [3], the human respiration caused a strain range from 0.05% to 10% [4], and the bending behaviors of the finger, elbow, and foot caused a strain change achieving >40% [5]. For stress, its change range was from <0.1 [6] (generated from gentle touch) to >100kPa [7,8] (generated from human body weight ). Thus, a device with a wide linear range and wide stress and strain detection range is required [9,10]. However, the current CNT-PDMS-based flexible devices aimed to achieve high piezoresistive sensitivity for strain measurement—the percolation threshold of the CNT-PDMS composite (the weight ratio of CNT and PDMS=2%~4%)—which limits their application in the stable output of large strain (>30%) [11].

The CNT-PDMS composite material exhibits excellent electromechanical, mechanical [12], electrical, and biocompatible properties [13,14,15]; thus, types of sensors including piezoresistive sensors, capacitive sensors [16], and piezoelectric sensors made from the CNT-PDMS composite are widely applied in medical diagnosis [17,18], health examination [19,20,21], and energy storage [22]. Among them, CNT-PDMS composite based piezoresistive sensors utilize the principle that the resistance of CNT-PDMS would change when the strain/deflection impacts the CNT-PDMS device. It has been reported that the CNT-PDMS composite in different weight ratios would produce a change in the electromechanical properties (e.g., piezoresistivity), mechanical properties (e.g., Young’s modulus and stiffness), and electrical properties (e.g., conductivity) of the CNT-PDMS composite. Thus, it would change the performance in sensitivity, linear range, and detection limit of the sensors made by the CNT-PDMS composite. These sensors have been widely developed through various fabrication techniques, such as lithography [23], casting molding [24,25], screen printing [26], micro-contact printing (transfer printing) [27], a deep etching process, and vacuum filtration [28,29]. For example, soft-lithography was commonly used to prepare a polysaccharide template for the CNT-PDMS-based capacitance-type sensor. The polysaccharide template plays the role of a sacrificial layer for meeting the requirement of the CNT-PDMS composite with a porous structure. The different diameters of porosity would directly affect the capacitance of the device (from 6.4 × 10^−11^ F to 2 × 10^−10^ F) [30,31]. In another example, a deep ion etching process was used to fabricate piezoelectric sensors. The sensor commonly consists of a CNT-PDMS-based electrode and another metal-based electrode [20,32]. When the pressure was applied or sliding occurred between the two electrodes, the balance of the charge distribution was broken, and the voltage pulse would be produced [33]. Some studies revealed that piezoelectric sensors made by the different weight ratios of CNT-PDMS composites can achieve different magnitudes of voltage output (from 0.8 to 3.1 V) [34].Thus, the weight ratio of CNT and PDMS would directly affect the electromechanical properties of the CNT-PDMS composite, affecting the performance of a sensor. Compared with previous results, when the weight ratio of the CNT-PDMS composite approaches the value of the percolation threshold, the change of electrical conductivity approaches the maximum value, and the piezoresistive sensitivity for the strain approaches the maximum response [11,35]. However, this limits the linear range between resistance change and the strain of the sensor using this weight ratio, and the introduced electronic noise will immerse the detected resistance change. Thus, a detailed understanding of the electromechanical, mechanical, and electrical properties of the CNT-PDMS composite formed in different weight ratios is essential.

CNT-PDMS composites can be prepared by methods of dry blending [10,36,37] and organic solvent methods [7,38,39]. The organic solvent method could uniformly disperse CNTs into PDMS. For example, both PDMS and CNT can be uniformly dissolved in chloroform, and the CNT–chloroform solution could not be precipitated within 70 hours. SEM showed that the diameter of the CNT cluster made by the organic solvent method was less than 3 μm, and the percolation threshold for strain sensitivity appeared at 1~2 wt% [40]. For the CNT-PDMS composites made by the dry blending method, the diameter of the internal cluster is commonly larger than 10 μm [41], and the percolation threshold for strain sensitivity appeared at 2~3 wt%. Although the electromechanical and other properties of CNT-PDMS composites are lower than those of CNT-PDMS made by the organic solvent method, the organic solvents used are all toxic and harmful substances. For example, LC_50_ of rats in chloroform was 117–125 mg/L air and it could produce Poisonous phosgene (COCl_2_), which has potential safety hazards [42].

In this work, we prepared the CNT-PDMS composite samples with different CNT weight ratios from 1 to 10 wt% using the dry blending method. The electromechanical testing for each weight ratio CNT-PDMS sample was performed including the piezoresistive properties by a universal testing machine and a custom-made tensile instrument, and the linear range, sensitivity, and stability of the piezoresistivity were also analyzed. The mechanical properties including the stress–strain, elastic modulus, and other mechanical properties, were also tested. For electrical properties, we detailly tested the cyclic voltammetry curve, conductivity, and impedance of the samples formed in each weight ratio. The results could be a data sheet for the CNT-PDMS composite and provide a reference for designing a CNT-PDMS-based sensor in the future. Furthermore, we used a suitable weight ratio (8%) of CNT-PDMS composites for measuring the large strain caused by the movement of different parts of the body.

## 2. Methods

### 2.1. Materials 

The MWCNTs which we used in experiments all came from Suzhou Tanfeng China (purity >97%, outer diameter: 8–15 nm, length: 10–20 μm). The used sylgard 184 PDMS was purchased from Dow Corning. A universal testing machine we used was from Shimadzu AGS-X5KN. The SEM image was obtained by Hitachi Regulus8220. The electrochemical workstation we used was Metrohm Autolab PGSTAT302N from Switzerland. The precise multimeter which we used was Agilent 34972A (Agilent Technologies, Santa Clara, CA, United States). The stretching instrument was custom-made by us.

### 2.2. Preparation of Different Weight Ratio CNT-PDMS Composites

**Figure 1:**
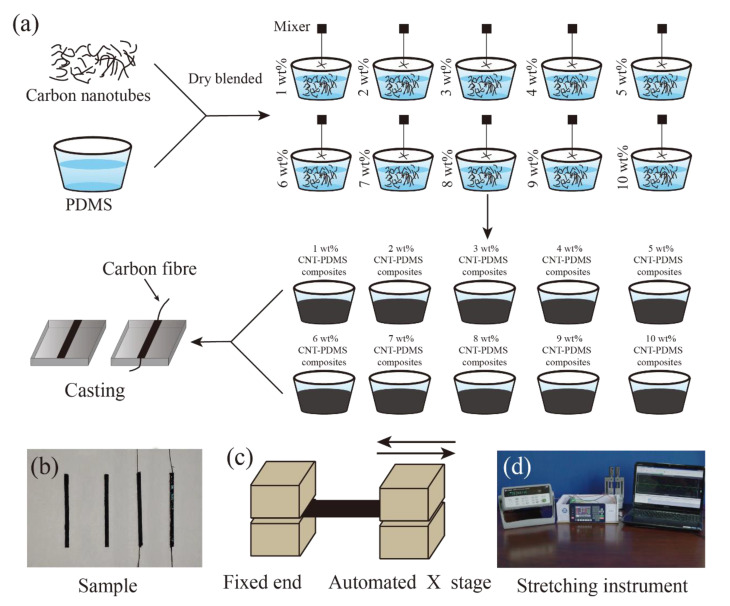
(**a**) A schematic illustrating the preparation procedure of the CNT-PDMS composites sample; (**b**) image of CNT-PDMS composites samples. The right side is the sample with carbon fiber embedded at both ends. The same process was used to prepare standard samples without embedding carbon fiber. (**c**) The schematic for the cyclic stretching test. (**d**) The custom-made platform for stretching samples of CNT-PDMS composites.

To study the effect of CNT content on the properties of the CNT-PDMS composite, we used the dry blending method to disperse CNT. First, we added 0.1 g CNT to 9.9 g PDMS (without PDMS curing agent), after blending at a low speed (150 r/min) for 24 h, 1wt% CNT-PDMS composites (without PDMS curing agent) were prepared. The prepolymer CNT-PDMS composites with a weight ratio of 2, 3, 4, 5, 6, 7, 8, 9, and 10 wt% were, respectively, prepared by the same procedure, as shown in Figure 1a.

To obtain comparable results in each mechanical and electrical testing, a standard acrylic mold (3 × 3 × 30 mm) was designed to fabricate samples with a uniform dimension. Vacuum treatment of the prepolymer CNT-PDMS composites was placed in the vacuum pressure chamber (<40 Pa) for 30 minutes [43], and then PDMS curing agent was added into the prepolymer CNT-PDMS composites with the weight ratio of the prepolymer CNT-PDMS composite and the curing agent 10:1. After that, the mixed composite was poured into the 3 × 3 × 30mm mold. The carbon fiber was embedded into both ends of the model. The embedding depth was about 0.5 mm. Finally, an oven was employed to solidify the mixed CNT-PDMS composite at a temperature of 70 °C for 5 h. The CNT-PDMS composites samples are shown in Figure 1b.

### 2.3. Measurement of Electrical Properties of the CNT-PDMS

The cyclic voltammetry (CV) of standard samples was measured from 0–4 V at a scanning speed of 50 mV/s using an electrochemical workstation (Metrohm Autolab PGSTAT302N, Herisau, Switzerland). The CV curve could be used to calculate the resistance (*R*_0_) of each sample. The electrical conductivity of the composite was calculated by the following formula:

$$\sigma =\frac{l}{SR_{0}}$$

where *S* is the sample of the cross-sectional area (m^2^), *l* is the length of the sample (m), *R*_0_ is the sample resistance (Ω), and σ is the sample of conductivity (S/m). Each CV test was measured four times, and four CNT-PDMS composites of each weight ratio were measured. 

Electrochemical impedance spectroscopy (EIS) was also performed using the electrochemical workstation (Metrohm Autolab PGSTAT302N, Herisau, Switzerland) with a frequency range of 0.1–10 kHz.

### 2.4. Morphological Characterization of the CNT-PDMS Composite

Field emission scanning electron microscopy (Hitachi Regulus8220, Tokyo, Japan) was performed to observe the cross-section microstructure of the CNT-PDMS composites in different weight ratios. Samples needed to undergo gold-coating treatment before the scanning. From the morphology, we could obtain the distribution of the CNT cluster in PDMS. 

### 2.5. Tensile Testing of the CNT-PDMS Composite

A universal testing machine (Shimadzu AGS-X5KN, Kyoto, Japan) was used to conduct tensile testing on standard samples without embedded carbon fibers. This measurement can determine the mechanical properties of the CNT-PDMS composite from the load-displacement curves.

### 2.6. Piezoresistive Measurement of the CNT-PDMS Composite

The piezoresistive properties of the CNT-PDMS composite were used by the CNT-PDMS samples with carbon fiber. Both ends of the CNT-PDMS composite were clamped into a custom-made stretching instrument (Figure 1c). The strain range (0% to 5%) was repeatedly stretched 15 times at a constant speed of 3 mm/s (the schematic is shown in Figure 1d); meanwhile, the resistance value of the sample was recorded with a precise multimeter (Agilent 34972A, Santa Clara, CA, United States). Similarly, different strain ranges (0% to 10%, 0% to 15%, 0% to 20%, 0% to 25%, 0% to 30%, 0% to 35%, 0% to 40%) were used to conduct the piezoresistive measurements. Six samples were tested for each weight ratio CNT-PDMS composite. The above experiments were carried out at room temperature (26 °C).

## 3. Results and Discussions

### 3.1. Electrical Properties of CNT-PDMS in Different Weight Ratios

#### 3.1.1. Electrical Resistance of CNT-PDMS in Different Weight Ratios

**Figure 2:**
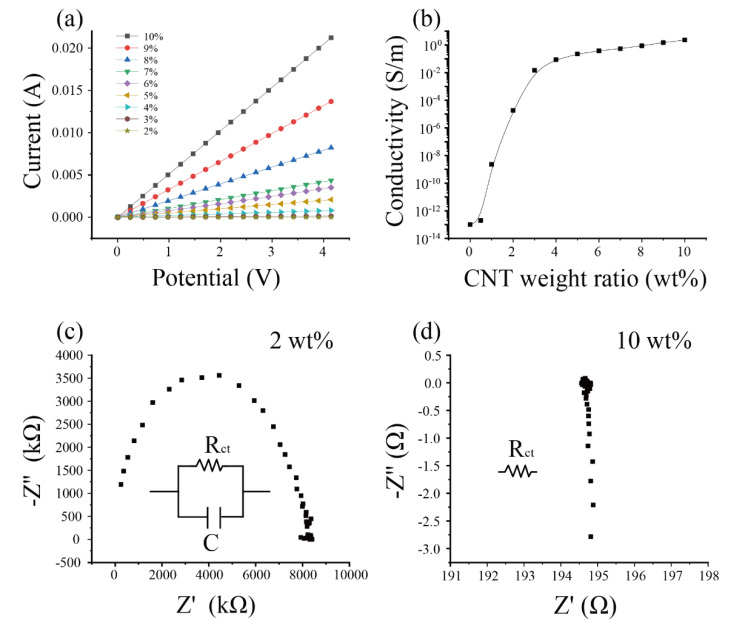
Electrical properties of CNT-PDMS, (**a**) the V-I curves of the CNT-PDMS composite with different weight ratios (wt%), (**b**) the relationship between the electrical conductivity of the CNT-PDMS composite with different weight ratios, (**c**) the impedance analysis of the 2 wt% CNT-PDMS composite and its equivalent circuit model, (**d**) the impedance analysis of the 10 wt% CNT-PDMS composite.

The electrical resistance of the CNT-PDMS composite is the prerequisite for determining the piezoresistive performance of the CNT-PDMS composite [14]. To obtain the electrical resistance of the CNT-PDMS composite, we measured the CV curves of the different weight ratio CNT-PDMS composites. Figure 2a shows that all samples exhibited linear CV curves during the scanning range from 0 to 4 V. From the CV curve, the electrical resistances of the CNT-PDMS composites could be calculated. With the increase in CNT weight ratio, the resistance of the CNT-PDMS composite samples reduced. For example, the resistance of the 2 wt% CNT-PDMS composite was greater than 4.0 × 10^7^ Ω. The resistance of the 3 wt% CNT-PDMS composite was 3.6 × 10^4^ Ω. However, when the weight ratio of CNT-PDMS increased to 10 wt%, the resistance of the CNT-PDMS composite reduced to around 200 Ω.

#### 3.1.2. Electrical Conductivities of CNT-PDMS in Different Weight Ratios

Compared with the electrical resistance, the electrical conductivity is independent of the dimension of the inspecting material. Figure 2b shows that the relationship between the electrical conductivity of CNT-PDMS and the weight ratio of CNT-PDMS, which revealed a sharp increase between 1 and 3 wt%, and there was a gradual increase in conductivity with a mild slope above 3 to 10 wt%. Compared with 2 wt%, the electrical conductivity of the 3 wt% CNT-PDMS composite sharply increased by over 1000 times in magnitude (<3 × 10^−5^ S/m vs. 0.015 S/m). When the weight ratio of CNT-PDMS achieved 6 wt%, the electrical conductivity of the CNT-PDMS composite tended to be satiated. In this CNT-PDMS composite made by the dry blending method, the sharp increase corresponded to percolation threshold which was 1–3 wt% (close to 2 wt%, which is consistent with the literature) [44,45]. Compared with carbon black and other metal particles, the CNT composite has the lower percolation threshold [46]. These results may be related to the tube shape of CNT and its larger length–diameter ratio. The piezoresitive performance of the CNT-PDMS composites (weight ratio <3 wt%) was out of the measurable range of our instrument (>40 MΩ, Agilent 34972A, Santa Clara, CA, United States).

#### 3.1.3. Electrical Impedance of the Different Weight Ratio the CNT-PDMS Composite in Alternating Current Mode 

Electrical impedance spectroscopy (EIS) was employed to capture the frequency responses of CNT-PDMS in different weight ratios. The Nyqusit plot was an important method for extracting impedance elements from the frequency response [30]. Figure 2c shows that a semicircle curve is the frequency response of the 2 wt% CNT-PDMS composites. This demonstrates that the parallel of an electrical resistance and a capacitance [47] can represent the electrical equal circuit to the 2 wt% CNT-PDMS composites. With CNT-PDMS weight ratio increasing to 4% and other high values, the frequency response changed to be a vertical line. This revealed that the frequency response can be regarded as an electrical resistance. Figure S1a–g shows the same electrical impedance spectroscopy of the CNT-PDMS composites in different weight ratios (3, 4, 5, 6, 7, 8, and 9 wt%).

### 3.2. Morphologies of the CNT-PDMS Composites

**Figure 3:**
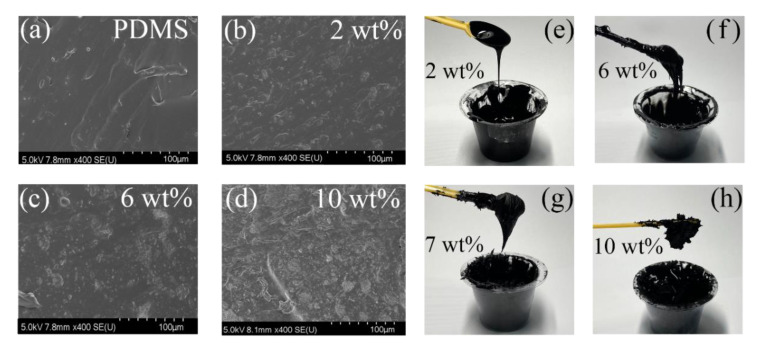
SEM image of CNT-PDMS composites with different weight ratio and photograph of CNT-PDMS composites; (**a**) SEM image of PDMS; (**b**) SEM image of 2 wt% CNT-PDMS composites; (**c**) SEM image of 6 wt% CNT-PDMS composites; (**d**) SEM image of 2 wt% CNT-PDMS composites; (**e**) photograph of CNT-PDMS composites at 2 wt%; (**f**) photograph of CNT-PDMS composites at 6 wt%; (**g**) photograph of CNT-PDMS composites at 7 wt%; (**h**) photograph of CNT-PDMS composites at 10 wt%.

There are two mechanisms for explaining the conductivity of CNT-PDMS composites: (1) the interconnections among adjacent CNTs can connect to the conductive pathway [48,49,50]; (2) the gap among the adjacent CNTs is less than 10 nm; therefore, the quantum tunneling effect can be activated. These two effects jointly determine the conductivity of CNT-PDMS composites [51]. Figure 3a–d and Figure S2 show the cross-section SEM images of the composites in different CNT-PDMS weight ratios (from 2 to 10 wt%). Few CNT clusters with diameters from 1 to 10 μm existed in the 2 wt% CNT-PDMS composites. The distances between the CNT clusters were all more than 1 μm, which revealed that the conductive and piezoresistive performance would be very weak in this weight ratio. With the weight ratio achieving >3 wt%, more CNT clusters were dispersed in PDMS. Full connections of adjacent CNTs were connected to form a conductive network in the composites, and the distance between adjacent CNTs became smaller, leading to the sharp increase in the conductivity of the composite materials. Macroscopically (Figure 3e–h), CNT-PDMS composites with a weight ratio from 1 to 4 wt% are similar to pure fluid PDMS and can drop naturally under the effect of gravity. These CNT-PDMS composites were used as the sensors in the shape of sponge, aerosol, and thin film [7,28,30,50]. The CNT-PDMS with a weight ratio from 5 to 7 wt% were still fluid but with a high viscosity, which could be spun to be film at a speed of >1000 r/min [6,29]. The CNT-PDMS composites with a weight ratio from 8 to 10 wt% were similar to the gel without fluidity. By using the fabrication process of screen printing and casting, CNT-PDMS composites (weight ratio 8%~10%) can be used as flexible strain sensors [15,38,44,52].

### 3.3. Mechanical Properties of the CNT-PDMS Composites

**Figure 4:**
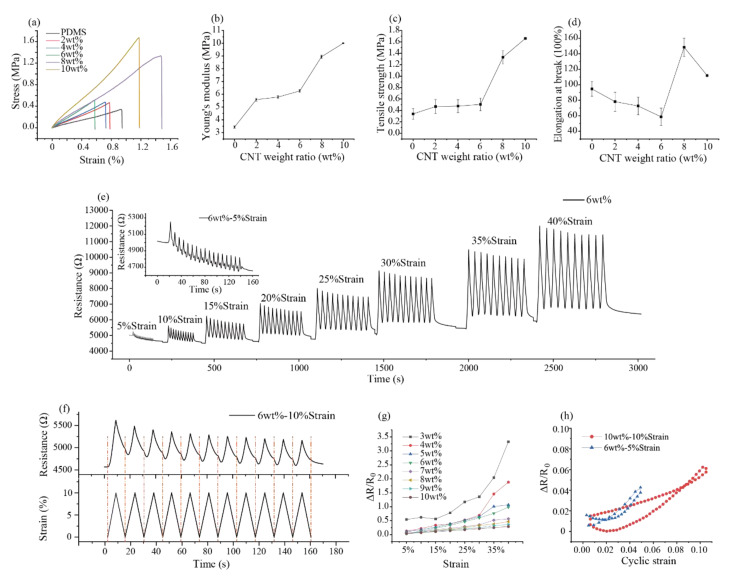
Mechanical properties and piezoresistive behavior of CNT-PDMS composites: (**a**) typical tensile stress–strain curves; (**b**) Young’s modulus; (**c**) tensile strength; (**d**) elongation at the break; (**e**) change in resistance upon the strain of the 6 wt% CNT-PDMS sample; (**f**) typical piezoresitive behavior of the CNT-PDMS composites sample (6 wt%), 10 strain; (**g**) resistance change of CNT-PDMS composites with different CNT weight ratios; (**h**) resistance during the stretching−releasing cycle.

To obtain the mechanical properties (e.g., Young’s modulus, tensile strength) of the CNT-PDMS, the stroke-load curve was measured by a universal testing machine. Figure 4a shows the stress–strain curves of the different weight ratio CNT-PDMS that was extracted from the measured stress–strain curve. The specific mechanical parameters including Young’s modulus, tensile strength, and elongation at the break could be calculated from the curves.

The slope of the stress–strain curve represents the Young’s modulus of the composites. Figure 4b shows that Young’s modulus of CNT-PDMS composites increased with the increase in CNT-PDMS weight ratio. The Young’s modulus of pure PDMS (without CNT) was 3.43 MPa, while the Young’s modulus of the 10 wt% CNT-PDMS composites was nearly triple that of the pure PDMS (10 vs. 3.34 MPa). This is because the Young’s modulus of CNT was larger than 1 TPa, and the tensile strength of CNT was larger than 1 GPa, which was much larger than the PDMS [53]. With the increase in weight ratio of CNT-PDMS, more and more CNTs in the composites were intertwined together; thus, the Young’s modulus of the composites enhanced. The maximum tensile strength of composites (Figure 4c and Table 1) had a similar trend to the Young’s modulus.

The elongation at the breaking point of the CNT-PDMS composites (Figure 4d and Table 1) decreased first and then increased with the increase in the weight ratio of CNT. The 8 wt% CNT-PDMS composites reached the maximum elongation. Moreover, the elongations of the sample in the weight ratio that we tested were all over 40%, which revealed that the CNT-PDMS could be used in large strain testing. The reason why 10 wt% CNT-PDMS composites have a greater tensile strength but a lower elongation rate than those of the 8 wt% CNT-PDMS composites is that although 10 wt% composites have more CNT interconnections within them, increasing their tensile strength causes stress concentration to occur in some CNT agglomerations, so they are more likely to be broken [54]. 

### 3.4. Piezoresistive Behavior of the CNT-PDMS Composites

When a certain tensile stress is applied to CNT-PDMS composites, the electrical conductivity value of the composites changes. This is also the reason that CNT-PDMS composites can be used to fabricate piezoresistive sensors. To provide detailed piezoresistive properties of CNT-PDMS in different weight ratios, the relationship between the tensile loading and the electrical resistance was changed. Figure 4e shows that the resistance of the 6 wt% CNT-PDMS composites cyclically changed with different tensile loadings, including strain ranges from 5%, 10%,15%, 20%, 25%, 30%, 35%, and 40%. Figure S3a–g shows the same piezoresistive testing of the CNT-PDMS composites in different weight ratios (3, 4, 5, 7, 8, 9, and 10 wt%). Each strain in the testing was repeatably performed for 11 cycles, which was used to verify the output stability of the CNT-PDMS composites, as in Figure 4f. And Figure 4h shows resistance change during the stretching−releasing cycle. During each strain cycle, the resistance of the CNT-PDMS composites increased gradually with the increase in the loaded strain, and the resistance of CNT-PDMS composites also decreased gradually with the decrease in the loaded strain. This is because the distance among the adjacent CNTs becomes larger when the composites material is stretched, and the previous CNT conductive pathway is be destroyed [55]. 

Because the inside conductive structure of the unstretched CNT-PDMS composites was unstable, the initial resistance of the CNT-PDMS composites gradually decreased and eventually stabilized with the ongoing cyclic strain (Figure 4e). We calculated the resistance changes ($\Delta R/R_{0}$) of the CNT-PDMS composites at different tensile strains in different CNT-PDMS weight ratios, as shown in Figure 4g and Table 2. Overall, for the same CNT-PDMS composites, the resistance change ($\Delta R/R_{0}$) rate increased with the increase in the strain. For CNT-PDMS with different weight ratios, the resistance changes ($\Delta R/R_{0}$) decreased with the increase in CNT-PDMS weight ratio under the same strain. The sensitivity (gauge factor, GF) could quantitatively evaluate the property of the such piezoresistive response:

$$\mathrm{GF}=\frac{\Delta R/R_{0}}{\Delta l/l_{0}}$$

where the GF is sensitivity, ΔR is resistance change of CNT-PDMS (Ω), initial resistance value is $R_{0}$(Ω), Δl is the change in length of the sample (mm), and $l_{0}$ is the initial length of the sample (mm). 

Figure 4g shows that the relationship between resistance change and strain is not linear. Therefore, we calculated the linear ranges between resistance change rate and the strain for each weight ratio CNT-PDMS composite, as well as the sensitivity, which is shown in Table 2. In our measurements, the piezoresistive response of the CNT-PDMS composites (1 and 2 wt%) could not be measured due to their low conductivities. The 3 wt% PDMS composites had a high sensitivity within the linear piezoresistive linear range; however, its linear range was narrowed (3 wt%: 15–25% vs. 8 wt%: 0–40%). In addition, the output resistance change was unstable during the repeated cyclic stretching. The piezoresistive linear range increased from 30% to 35% strain with the weight ratio increasing from 5–7 wt%. The piezoresistive linear range increased to 40 %, and the linear fitting coefficient achieved 0.99 when the weight ratio of CNT-PDMS was in the 8–10 wt% range. Hence, the 8wt% CNT-PDMS composite was the most suitable for strain measurement, which had a favorable sensitivity (better than 9–10 wt%), excellent linear range (better than 3–7 wt%), and good stretchability(better than 10 wt%). And it was also better than those of previously studied CNT-PDMS strain sensors (see Table S1 in the Supplementary document). Therefore, 8 wt% was selected to produce a flexible device for measuring wide strain behaviors of the human body in the following experiments.

### 3.5. Application of the CNT-PDMS Composites

**Figure 5:**
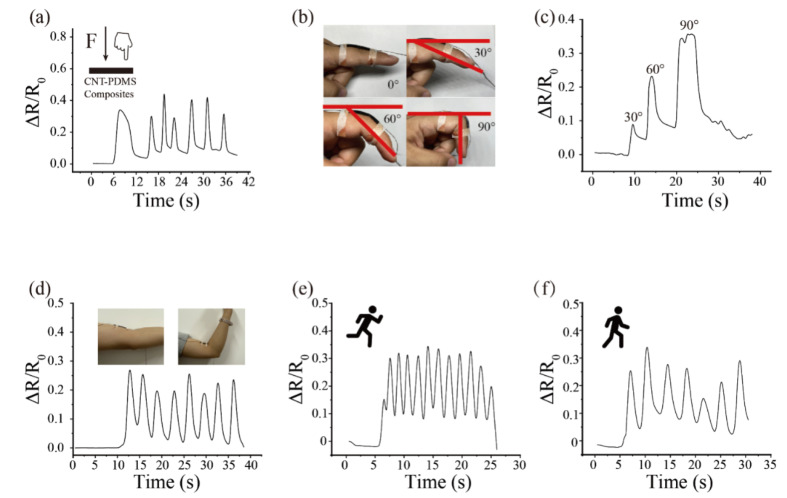
Application of the CNT-PDMS composites sensor (**a**) resistance changes by finger touch CNT-PDMS composites sensor, (**b**–**c**) resistance changes when the fingers are bent to different degrees, (**d**) CNT-PDMS composites sensor to monitor the joint and muscle movements. (**e**–**f**) resistance changes during running and walking.

To demonstrate an application in the wide strain and stress range measurement of the 8 wt% CNT-PDMS composites, a flexible strain sensor was developed for monitoring the human body. A volunteer’s finger with a force of 4.9 N was applied to the middle of the sample (strain = 25%), as shown in Figure 5a, the resistance change and the duration in each press were captured. In addition, the strain sensor was used to detect different angles of finger bending (30, 60, and 90°) and the contractions of the biceps muscle. The electrical resistance varied with different bending angles of the finger. When the finger was bent at 90° (strain = 30%), the electrical resistance change could achieve 0.35 (Figure 5b–c). Moreover, the resistance change of CNT-PDMS can also be detected during bicep contraction (strain = 15%). Finally, the strain sensor was glued to the heel of the shoe to record the electrical resistance signals caused by the stress of the volunteer (60 kg, stress = 15 kPa) during running and walking (Figure 5e–f); besides this, the frequencies of running and walking could be clearly distinguished.

## 4. Conclusions

In this study, the electromechanical, mechanical, and electrical properties of the CNT-PDMS composite in different weight ratios (0–10 wt%) were determined. The 3 wt% CNT-PDMS composite was found to exhibit the highest sensitivity to the strain measurement, and the narrowed linear range (15%–25%) and large background noise limit its application in wide strain/stress measurement. With the increase in CNT-PDMS weight ratio, the mechanical properties and linear range for strain were significantly improved. In summary, the 8 wt% CNT-PDMS composite exhibits a wide piezoresistive linear range (0%–40%) and a comparable sensitivity (gauge factor = 1.21), which is suitable for wide strain/stress measurement.

## Figures and Tables

**Table 1:** Mechanical properties of composites.

Sample	Tensile Strength (MPa)	Young’s Modulus (MPa)	Elongation at the Break (%)
PDMS	0.339	3.434	94.58
PDMS-CNT (2 wt%)	0.467	5.583	78.17
PDMS-CNT (4 wt%)	0.475	5.783	72.59
PDMS-CNT (6 wt%)	0.505	6.262	58.6
PDMS-CNT (8 wt%)	1.333	8.938	148.35
PDMS-NT (10 wt%)	1.669	10	111.8

**Table 2:** Pressure-sensitive properties of CNT-PDMS composites: materials.

CNT Content	Linear Piezoresistive Range	Sensitivity in the Linear Range	R^2^ (in the Linear Range)	Initial Resistance R_0_ (Ω)	Conductivity (S/m)
1 wt%	——	——	——	——	<10^−9^
2 wt%	——	——	——	>40 MΩ	<3 × 10^−5^
3 wt%	15–25%	6.086	0.9737	35,682	0.0147
4 wt%	0–30%	2.1793	0.9741	9567.2	0.086
5 wt%	0–30%	2.1689	0.9867	3280.1	0.223
6 wt%	0–30%	1.5166	0.9789	8770.4	0.3774
7 wt%	0–35%	1.4321	0.9578	1395	0.5451
8 wt%	0–40%	1.2097	0.9921	529.14	0.8809
9 wt%	0–40%	0.9119	0.9937	492.06	1.4674
10 wt%	0–40%	0.7235	0.9997	344	2.275

## Supplementary Materials

The following are available online at https://www.mdpi.com/1424-8220/20/16/4523/s1. Figure S1: Impedance analysis of the CNT-PDMS composite with different CNT mass fraction; Figure S2: resistance change curves of different CNT mass fraction CNT-PDMS samples during stretching; Figure S3: SEM image of CNT-PDMS composites with different weight ratios; Table S1: performance comparison table of flexible sensors with different materials.
