# Supplementary material for: Optimized CNT-PDMS Flexible Composite for Attachable Health-Care Device
Source: Sensors (Basel). 2020 Aug 13;20(16):4523. doi: 10.3390/s20164523 (PMC7472186; doi:10.3390/s20164523)
Supplement: Supplementary file 1 [file sensors-20-04523-s001.pdf]

Supporting Information

Optimized CNT-PDMS Flexible Composite and its Application in Attachable Health-care Device

Jian Du †, Li Wang *,†, Yanbin Shi, Feng Zhang, Shiheng Hu, Pengbo Liu, Anqing Li and Jun Chen

Advanced Micro and Nanoinstruments Center (AMNC), School of Mechanical & Automotive Engineering, Qilu University of Technology (Shandong Academy of Sciences), Jinan 250353, China; 1043117031@stu.qlu.edu.cn (J.D.); syb@qlu.edu.cn (Y.S.); 201701040032@stu.qlu.edu.cn (F.Z.); 1043118164@stu.qlu.edu.cn (S.H.); pengbo@qlu.edu.cn (P.L.); akin@qlu.edu.cn (A.L.); chenjun@qlu.edu.cn (J.C.)

* Correspondence: liwang@qlu.edu.cn; Tel.: +86-0531-8963-1702; Fax: +86-0531-8963-1702

† Authors equally contributed

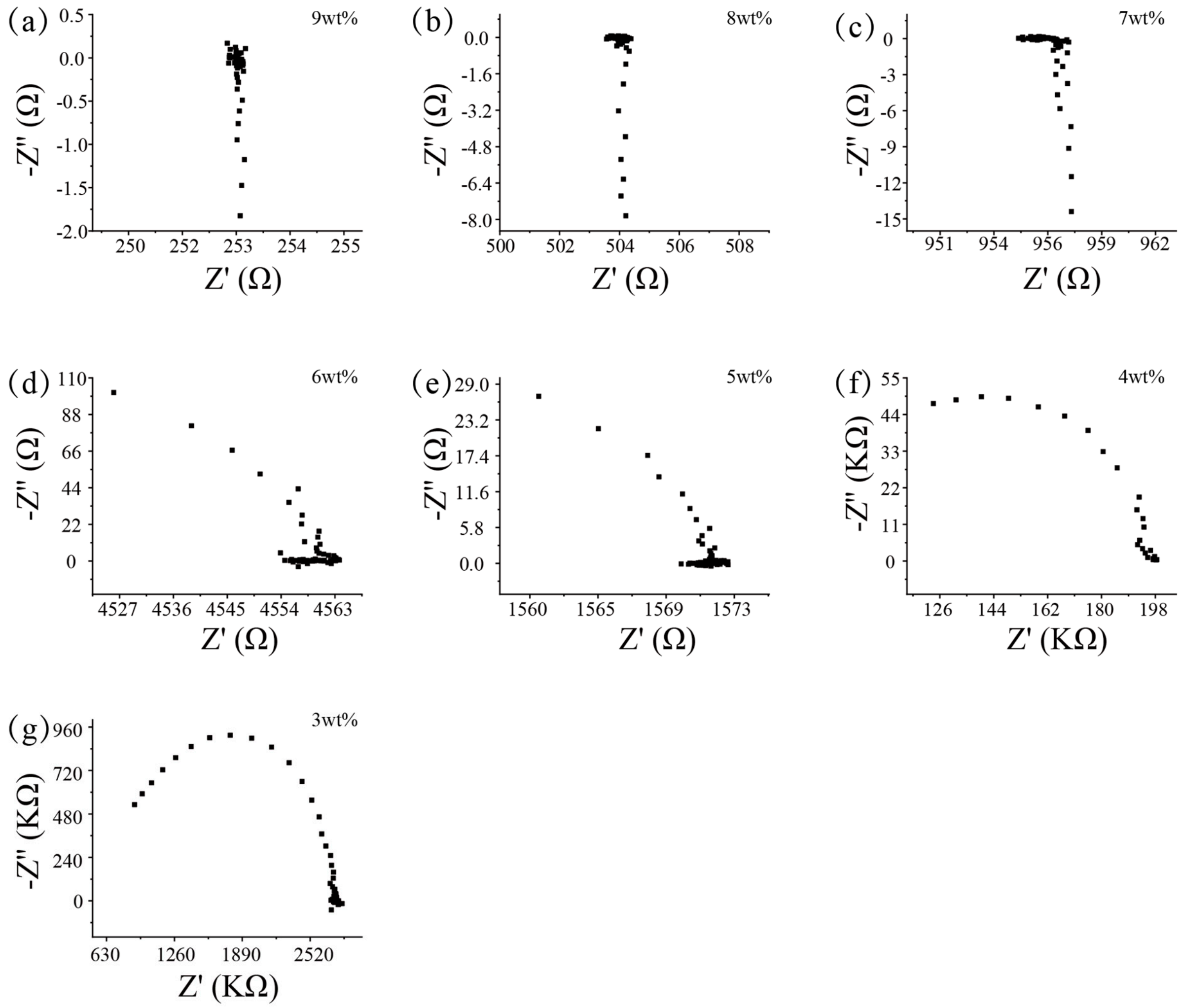

Figure. S1. (a-g) Impedance analysis of CNT-PDMS composite with different CNT mass fraction: (**a**) 9 wt% CNT-PDMS; (**b**) 8 wt% CNT-PDMS; (**c**) 7 wt% CNT-PDMS; (**d**) 6 wt% CNT-PDMS; (**e**) 5 wt% CNT-PDMS; (**f**) 4 wt% CNT-PDMS (**g**) 3 wt% CNT-PDMS.

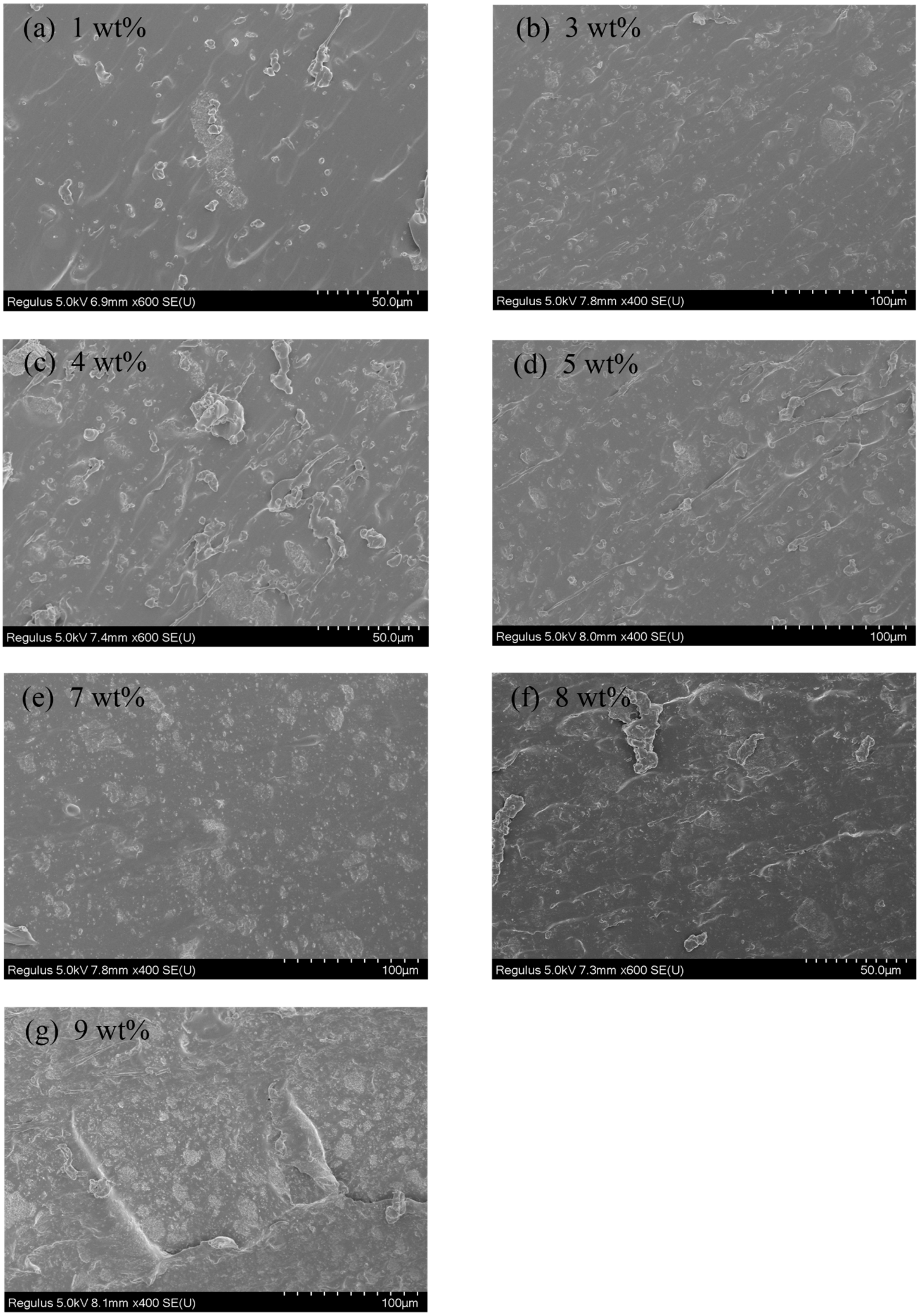
(a) 1 wt%
Regulus 5.0kV 6.9mm x600 SE(U)
50.0μm
(b) 3 wt%
Regulus 5.0kV 7.8mm x400 SE(U)
100μm
(c) 4 wt%
Regulus 5.0kV 7.4mm x600 SE(U)
50.0μm
(d) 5 wt%
Regulus 5.0kV 8.0mm x400 SE(U)
100μm
(e) 7 wt%
Regulus 5.0kV 7.8mm x400 SE(U)
100μm
(f) 8 wt%
Regulus 5.0kV 7.3mm x600 SE(U)
50.0μm
(g) 9 wt%
Regulus 5.0kV 8.1mm x400 SE(U)
100μm

Figure. S2.(a-g) SEM image of CNT-PDMS composites with different weight ratio: (**a**) 1 wt% CNT-PDMS; (**b**) 3 wt% CNT-PDMS; (**c**) 4 wt% CNT-PDMS; (**d**) 5 wt% CNT-PDMS; (**e**) 7 wt% CNT-PDMS; (**f**) 8 wt% CNT-PDMS (**g**) 9 wt% CNT-PDMS.

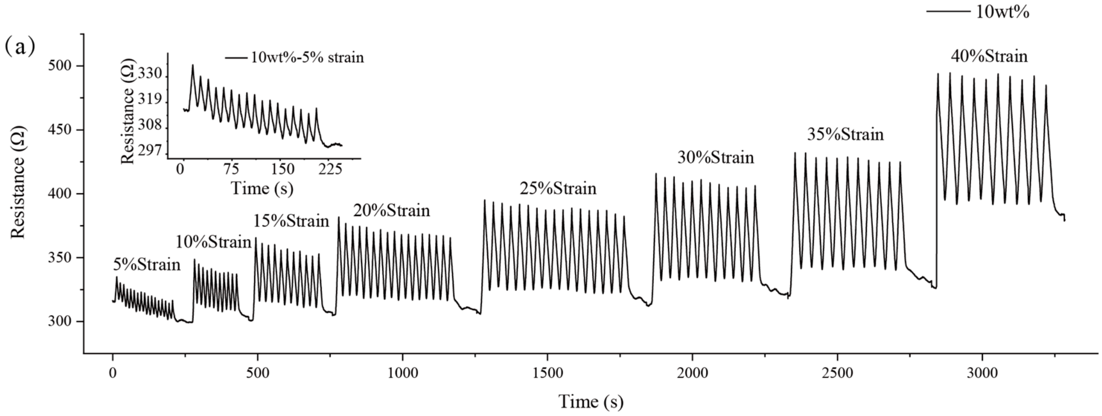

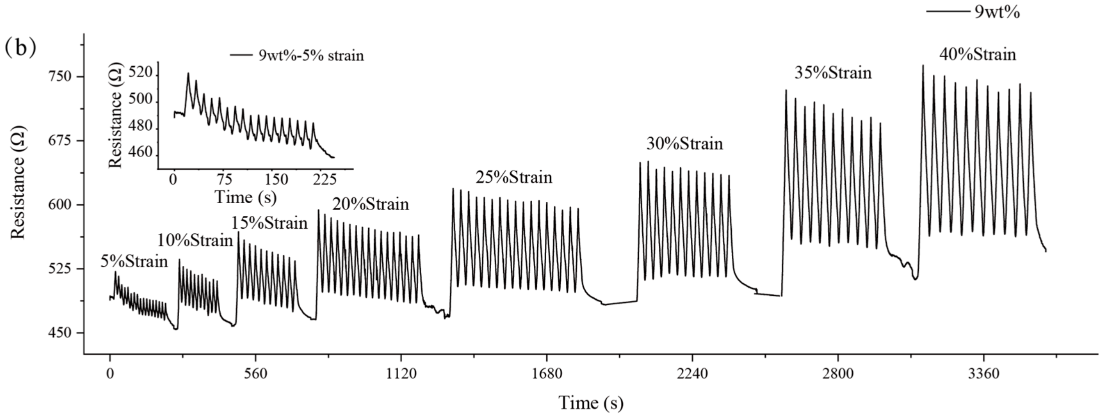

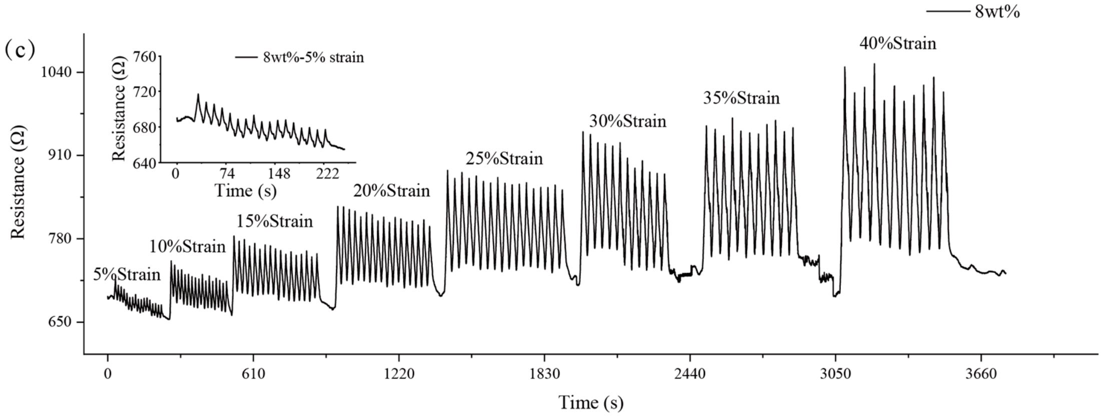

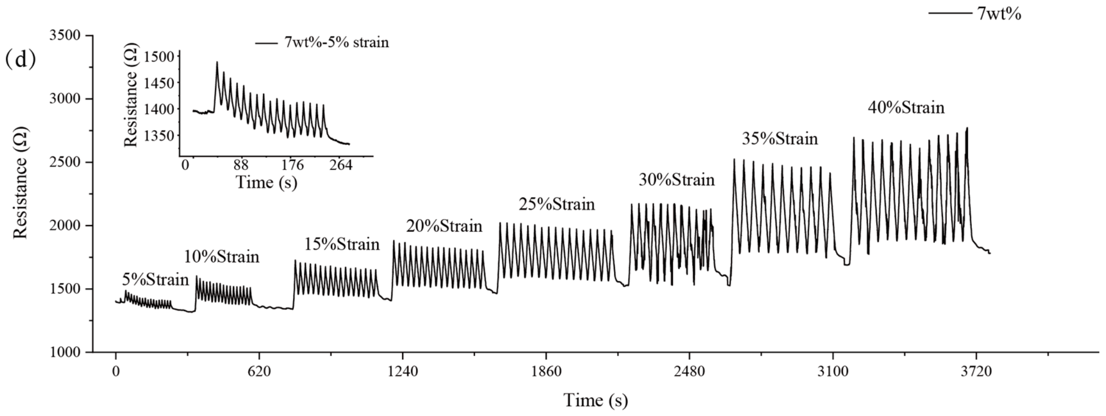
(d)
7wt%
7wt%-5% strain
Resistance (Ω)
Time (s)
5%Strain
10%Strain
15%Strain
20%Strain
25%Strain
30%Strain
35%Strain
40%Strain

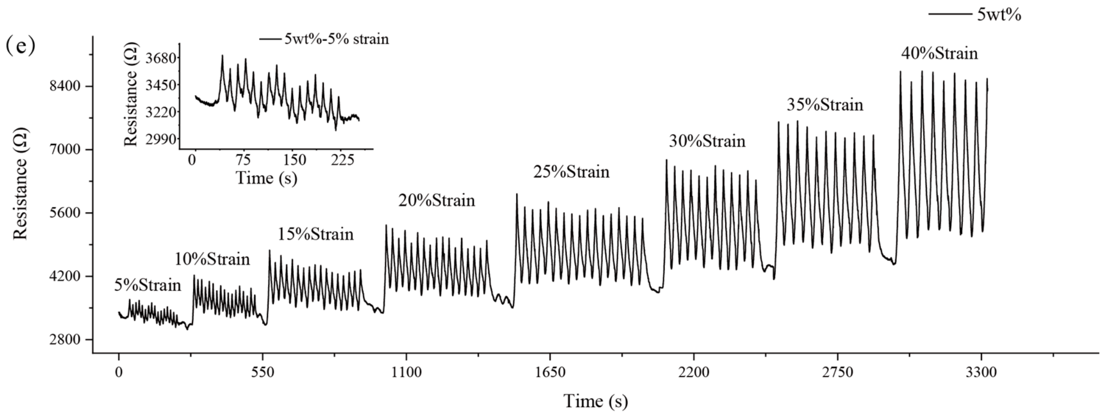
(e)
5wt%
5wt%-5% strain
Resistance (Ω)
Time (s)
5%Strain
10%Strain
15%Strain
20%Strain
25%Strain
30%Strain
35%Strain
40%Strain

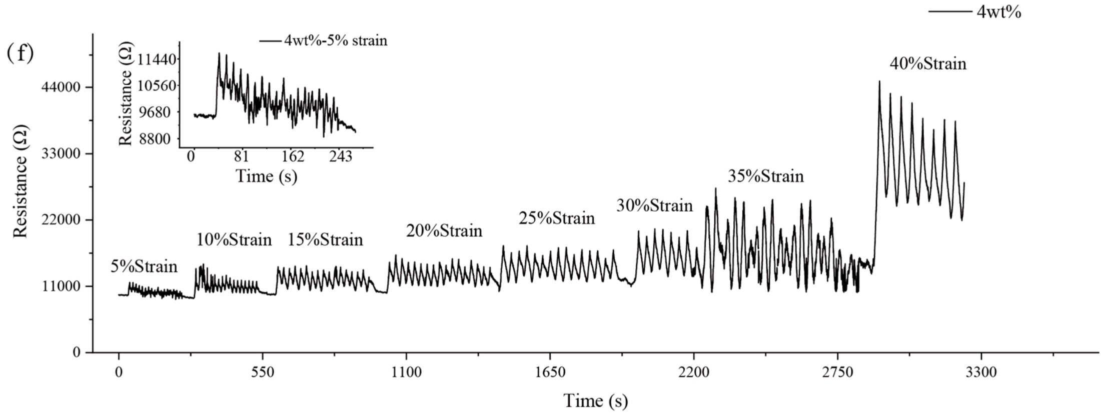
(f)
4wt%
4wt%-5% strain
Resistance (Ω)
Time (s)
5%Strain
10%Strain
15%Strain
20%Strain
25%Strain
30%Strain
35%Strain
40%Strain

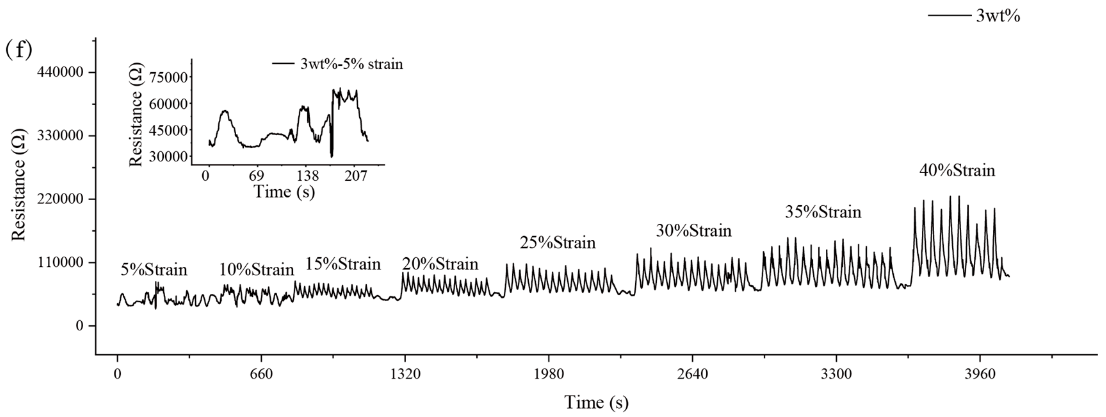

Figure. S3. (a-g) resistance change curves of different CNT mass fraction CNT-PDMS samples during stretching: (**a**) 10 wt% CNT-PDMS; (**b**) 9 wt% CNT-PDMS; (**c**) 8 wt% CNT-PDMS; (**d**) 7 wt% CNT-PDMS; (**e**) 5 wt% CNT-PDMS; (**f**) 4 wt% CNT-PDMS (**g**) 3 wt% CNT-PDMS.

Table 1. Performance comparison table of flexible sensors with different materials.

Material	Blending method	Sensitivity/ Gauge factor	Linear range	Toxicity	Ref.
15wt% CNT/ETC-PTHF	Organic solvent method	Max. 8900	Not shown	Not sure	[1]
7% MWCNT/PDMS	Organic solvent method	5 - 9	0% - 40% strain	little pentane left in the mixture	[2]
CB-TPU* (25 wt%)	evaporation organic solvent	2.56	0% - 1% strain	None	[3]
Graphene based on stretchable yarns	ayer-by-layer assembly technique	Not shown	0% - 150% strain	Not sure	[4]
TPU/CNT−CNC	Complex method	321	> 500% strain	Not sure	[5]
GnPs/epoxy**	ultrasonic and the ball mill mixing	22.54	0% - 1.2% strain	Not sure	[6]
This work (8%wt CNT-PDMS)	dry blending	1.2097	0% - 40% strain	Good biocompatibility	-

*CB-TPU = Carbon black nanoparticles - Thermoplastic polyurethane

**GnPs/epoxy = graphene platelets /epoxy

References:

1. Lind, J.U.; Busbee, T.A.; Valentine, A.D.; Pasqualini, F.S.; Yuan, H.; Yadid, M.; Park, S.; Kotikian, A.; Nesmith, A.P.; Campbell, P.H.; et al. Instrumented cardiac microphysiological devices via multimaterial three-dimensional printing. *Nat. Mater.* **2017**, *16*, 303–308.
2. Park, J.J.; Hyun, W.J.; Mun, S.C.; Park, Y.T.; Park, O.O. Highly Stretchable and Wearable Graphene Strain Sensors with Controllable Sensitivity for Human Motion Monitoring. *ACS Appl. Mater. Interfaces* **2015**, *7*, 6317–6324.
3. Wang, Y.; Mi, H.; Zheng, Q.; Zhang, H.; Ma, Z.; Gong, S., Highly stretchable and sensitive piezoresistive carbon nanotube/elastomeric triisocyanate-crosslinked polytetrahydrofuran nanocomposites. *J. Mater. Chem. C* **2016**, *4*, 460–467.

4. Fu, X.; Ramos, M.; Al-Jumaily, A.M.; Meshkinzar, A.; Huang, X. Stretchable strain sensor facilely fabricated based on multi-wall carbon nanotube composites with excellent performance. *J. Mater. Sci.* **2019**, *54*, 2170–2180.
5. Zhu, L.; Zhou, X.; Liu, Y.; Fu, Q. Highly Sensitive, Ultrastretchable Strain Sensors Prepared by Pumping Hybrid Fillers of Carbon Nanotubes/Cellulose Nanocrystal into Electrospun Polyurethane Membranes. *ACS Appl. Mater. Interfaces* **2019**, *11*, 12968–12977.
6. Lu, S.; Tian, C.; Wang, X.; Zhang, L.; Du, K.; Ma, K.; Xu, T. Strain sensing behaviors of GnPs/epoxy sensor and health monitoring for composite materials under monotonic tensile and cyclic deformation. *Compos. Sci. Technol.* **2018**, *158*, 94–100.
